# Analysis of P300 Evoked Potentials to Determine Pilot Cognitive States

**DOI:** 10.3390/s25196201

**Published:** 2025-10-07

**Authors:** Germán Rodríguez-Bermúdez, Benjamin Naret, Ana Rita Teixeira

**Affiliations:** 1 Centro Universitario de la Defensa, Universidad Politécnica de Cartagena, C/Coronel López Peña S/N, Base Aérea de San Javier, Santiago de La Ribera, 30720 Murcia, Spain; german.rodriguez@cud.upct.es (G.R.-B.); benjamin.naret@ecole-air.fr (B.N.); 2 Porto Polytechnic—ISEP and INED and GECAD and IEETA—Institute of Electronics and Informatics Engineering of Aveiro, 3810-193 Aveiro, Portugal

**Keywords:** P300, electroencephalogram, event related potential

## Abstract

The P300 evoked potential, recorded via electroencephalography, serves as a relevant marker of attentional allocation and cognitive workload. This work extracts and analyzes event-related potentials that reflect variations in the cognitive state of military pilots during a complex simulated flight scenario coupled with simultaneous mental arithmetic tasks. The experiment was conducted at the Academia General del Aire (Spain) with 14 military pilots using a high-fidelity flight simulator. The experimental protocol involved dynamic flight instructions combined with arithmetic tasks designed to elicit varying cognitive loads. The results revealed a significant decrease in P300 amplitude across successive sessions, indicating a progressive reduction in attentional engagement due to task habituation and increased cognitive automaticity. Concurrently, P300 latency for correct responses decreased significantly, demonstrating enhanced efficiency in cognitive stimulus evaluation over repeated exposure. However, incorrect responses failed to yield robust results due to an insufficient number of trials. These findings validate the use of P300 as an objective indicator of cognitive workload variations in realistic aviation contexts.

## 1. Introduction

Modern combat aircraft programs, such as the Future Combat Air System, underscore a fundamental fact of contemporary aviation: once airframes and engines reach a certain level of maturity, overall mission effectiveness is governed less by the platform’s physical limits than by the human operator’s cognitive margin [1]. Pilots must integrate dense streams of visual, auditory, and tactile information while executing time-critical decisions; if the neurocognitive system becomes saturated, situational awareness collapses and performance degrades long before the aircraft itself reaches aerodynamic or structural limits [2]. Understanding these limits and designing cockpits that respect them are therefore central themes in the discipline of human factors.

Electroencephalography (EEG) has emerged as a promising tool for probing those limits because event-related potentials (ERPs) map distinct stages of perception and decision on a millisecond scale. ERPs are time-locked voltage fluctuations in the EEG signal that are elicited in response to specific sensory, cognitive, or motor events, reflecting the synchronized activity of neuronal populations engaged during information processing [3]. Three prominent ERP deflections consistently emerge within the initial few hundred milliseconds after stimulus onset: the P100, N200, and P300 components.

The P100 is an early, positive-going ERP component that typically peaks around 100 milliseconds after the onset of a visual stimulus and is maximal over the occipital scalp. It originates in the visual cortex and reflects the brain’s initial registration of visual input. While it is largely considered an exogenous response, which means shaped by the physical properties of the stimulus, its amplitude is nonetheless sensitive to attentional modulation [4]. From a physiological perspective, the P100 serves as an index of the integrity and efficiency of early visual processing. In applied terms, a clearly identifiable P100 indicates that the stimulus was successfully perceived at the basic sensory level.

The N200 is a negative-going ERP component that emerges roughly 200 milliseconds after stimulus onset and is usually largest at fronto-central scalp sites, though it can also appear over occipito-temporal regions depending on task demands. It is classed as an endogenous response because its amplitude depends mainly on cognitive factors rather than stimulus physics. In particular, the N200 grows when the brain must discriminate between competing stimuli, detect a mismatch, or inhibit an automatic response [5]. Physiologically, the component is thought to arise from activity in anterior cingulate and fronto-parietal circuits that support early cognitive control. Functionally, the N200 marks the moment when perceptual analysis gives way to evaluative processes, making it a useful indicator of rapid attention allocation and conflict monitoring before full decision making unfolds [5].

The P300 is a broad, positive ERP component typically peaking around 300 milliseconds after stimulus onset, though it can range more widely between approximately 250 and over 400 milliseconds. Its amplitude is usually maximal at parietal–central electrode sites. Originally described by Sutton et al. (1965) [6], the P300 is one of the most extensively studied ERP components, initially identified as a prominent positive wave elicited when subjects detect rare or meaningful stimuli. Unlike earlier ERP components, the P300 amplitude is primarily determined by the psychological significance of the stimulus rather than its physical attributes, classifying it clearly as an endogenous, cognitive response. The P300 is particularly sensitive to attentional allocation: when cognitive resources are monopolized by a demanding primary task, the P300 response to a secondary stimulus tends to decrease, reflecting reduced availability of attentional capacity [7,8]. Moreover, its latency closely aligns with the duration of stimulus evaluation and decision-making processes, reflecting the cognitive time required to classify and respond to the event. Due to these characteristics, the P300 serves as a valuable, non-invasive marker of cognitive processing capacity, functioning effectively as a neural signature of attention and decision making [9]. Consequently, it has become a cornerstone of ERP research, extensively employed across various fields ranging from brain–computer interfaces to clinical diagnostics, as an objective indicator of cognitive state. Within the context of this study, the P300 will constitute the primary measure for assessing how pilots cognitively process task-relevant events under varying levels of mental workload [10,11,12].

There are several works that analyze the P300 response in an aeronautics context. Kramer, Sirevaag, and Braune [13] demonstrated systematic decreases in P300 amplitude with increasing difficulty of a simulated flight task, in which the participating subjects were required to discriminate between two tones differing in frequency and to perform an occasional overt response. Performance deterioration, reflected by deviations from assigned flight parameters, correlated with reductions in the P300. Similarly, Sirevaag et al. [14], using six military pilots and a visual and auditory oddball as a secondary task to increase the workload, reported decreased P300 amplitudes under increased communication load in a high-fidelity helicopter simulation. Fowler’s [15] research on instrument landings under varying conditions similarly emphasized the P300 as a reliable workload indicator. The P300 was elicited with auditory and visual “oddball” subsidiary tasks requiring the detection of infrequent tones or flashes of an artificial horizon.

Natani and Gomer [16] used an auditory oddball and revealed that pilots’ P300 responses to secondary auditory stimuli decreased in amplitude and increased in latency during challenging flight maneuvers, clearly reflecting a higher cognitive workload. Similarly, Theissen, Lay, and Stern [17,18] implemented an auditory and visual oddball and observed diminished P300 responses in electronic warfare officers during complex threat detection scenarios, confirming the ERP’s sensitivity to attentional resource allocation under demanding conditions.

Although not all simulator results have been uniformly consistent [19] (noted variability due to task differences and participant strategies), the overall findings consistently validate ERPs as real-time indicators of pilot workload. These simulator studies informed cockpit interface designs by identifying displays or systems imposing excessive cognitive loads, as indicated by reduced ERP amplitudes [20]. Moreover, early adaptive aiding concepts emerged from these studies, where EEG and ERP signals informed automated assistance during high workload scenarios. Notably, NASA’s studies, such as that by Prinzel et al. [21], demonstrated that real-time ERP monitoring could effectively guide automation, enhancing safety and performance in simulated missions. Recent studies have leveraged EEG to monitor pilot workload, detect overload or underload states, and trigger adaptive support [22,23,24].

There are also real flight experiments that confirm the P300 as a key indicator of workload. The feasibility of ERP-based cognitive monitoring during actual flight was significantly advanced by Hankins and Wilson [25], who successfully recorded EEG, including the P300, from certified pilots in real aircraft operations. Their findings underscored EEG’s sensitivity to workload variations, highlighting the practicality of EEG measures for operational environments. Subsequent real-flight studies, notably by Dehais et al. [18], confirmed these findings by demonstrating that P300 amplitude reliably decreased during high-workload flight segments, even under challenging recording conditions. Once again, this decrease does not indicate a lack of global attention, but rather a reallocation of cognitive resources toward primary task demands, at the expense of processing secondary stimuli typically associated with the P300. Thus, the results are in line with those obtained in high-fidelity simulators.

Dual-task paradigms combining memory or arithmetic challenges with an additional primary task, such as driving or interacting with a brain–computer interface, have demonstrated again that P300 attenuation is sensitive to divided attention and task prioritization [26,27].

ERP measures have also been instrumental in evaluating training effectiveness and situational awareness among pilots. Studies have indicated that expert pilots often exhibit faster and more robust P300 responses to critical stimuli compared to novices, reflecting more efficient cognitive processing and heightened situational awareness [21].

In summary, ERP-based cognitive monitoring has evolved significantly from controlled laboratory settings to realistic, complex simulator environments and actual flight conditions. ERP components, particularly the P300, consistently demonstrate sensitivity to workload fluctuations and attentional demands, providing reliable, objective metrics for pilot cognitive state assessment. These insights have profoundly impacted cockpit design principles and have catalyzed advancements toward adaptive aviation systems, potentially allowing aircraft to monitor pilot cognitive states in real-time and provide appropriate support [28].

Recognizing the value of such metrics for evidence-based interface design, researchers from Centro Universitario de la Defensa at the Spanish Air Force Academy (CUD-AGA) launched an initiative to generate a new EEG dataset recorded exclusively from military pilot trainees in a high-fidelity flight-simulation environment. Within that initiative, a modular protocol that combines realistic instrument flight maneuvers with secondary arithmetic memorization challenges has been developed. It induces three graded levels of mental workload and yields a rich corpus of EEG suitable for ERP analysis. The present study takes that corpus as its empirical foundation and, focusing on the dataset’s most demanding flight segment, analyzes how P300 dynamics reflect variations in the pilot’s cognitive state. Specifically, the analysis examines various types of stimuli embedded in the experimental design, with particular attention to the P300, differentiating between arithmetic memorization tasks when numbers appear on the screen and feedback processing under correct and incorrect answer conditions in arithmetic memorization tasks. This experiment is new, and it has not been performed before. There are no works that analyze military pilots’ P300 under this secondary task or compute and analyze the P300 when results are shown. The performance trends are then assessed across time and task parameters to interpret how cognitive processing evolves under sustained workload and to explore how human factors influence the pilot’s mental state in high-demand scenarios.

To systematically address the research objectives outlined above, this paper is structured as follows: Section 2 provides a comprehensive description of the origins of the EEG dataset utilized in this research and the detailed methodology. Section 3 shows the results computed in this work, Section 4 provides a discussion of the results, and, finally, Section 5 states the main conclusions.

## 2. Materials and Methods

This section provides a comprehensive description of the origins of the EEG dataset utilized in this research. It outlines the experimental environment, participant demographics, technical configurations, and detailed methodology, crucial for understanding and interpreting ERP dynamics within aviation human factors research.

### 2.1. Environment and Participants

The EEG dataset originated from an extensive experiment conducted at the Academia General del Aire (AGA) in San Javier, Murcia, Spain, as part of a collaborative internship between the French École de l’Air et de l’Espace (EAE) and the Spanish Centro Universitario de la Defensa (CUD). This partnership included participants from different stages of military pilot training. Specifically, the final sample consisted of fourteen right-handed male participants aged between 20 and 34, representing various stages of military pilot training: three third-year cadets with basic flight experience, ten fourth-year cadets with advanced training, and one experienced instructor.

Ethical considerations were rigorously maintained. This experiment was approved by the Ethical Committee of Universidad Politécnica de Cartagena, with the number CEI25-008.

### 2.2. Equipment

**Figure 1:**
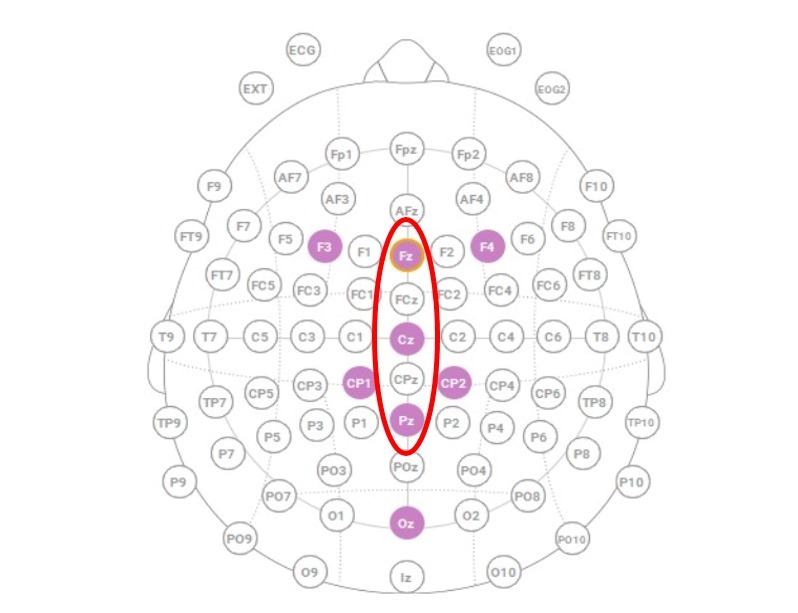
Electrode placement according to the 10/20 system and selected color electrodes for the analysis.

EEG data were collected using a Neuroelectrics Enobio8 headset from Barcelona, Spain, selected for its reliability and previous validation in aviation research contexts. This system incorporated eight active wet electrodes positioned according to the international 10-20 EEG electrode placement standard (Fz, Cz, Pz, Oz, F3, F4, CP1, CP2), optimized for capturing relevant cortical activity during flight and cognitive tasks; see Figure 1.

**Figure 2:**
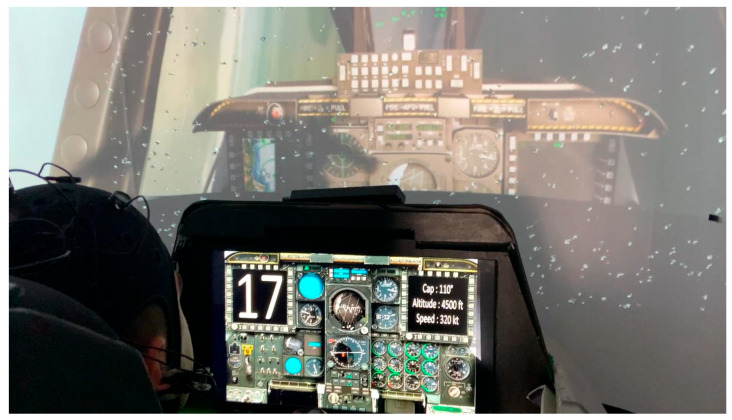
Picture of one volunteer inside the cockpit performing Module 3.

The headset was wirelessly connected via Bluetooth, allowing flexible usage during simulation, with data sampled at 500 Hz. An immersive flight simulation environment was constructed around a realistic cockpit replica of the A-10C Thunderbolt, integrated with Digital Combat Simulator (DCS) software and Immersive Display Pro. See Figure 2.

This configuration delivered a high-fidelity simulation, closely replicating actual cockpit operations and flight characteristics. The experiment employed multiple software tools to ensure experimental precision, effective EEG signal acquisition, and initial data analysis, including OpenVIBE version 1.3.0, MATLAB R2023a, complemented by EEGLAB version 2022.1, and DCS 2.9.3.51704.

### 2.3. Experiment Protocol

**Figure 3:**
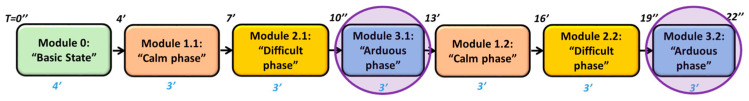
Experimental module sequence and timeline.

The experimental protocol was structured into distinct modules designed to systematically increase cognitive workload. Each module represented graduated complexity levels to rigorously assess cognitive workload and attentional demands. Figure 3 shows the sequence of modules, the timing starting in T = 0 and finishing in T = 22′, and the duration of each module at the bottom. The system presents Module 0 and, after that, it repeats Modules 1, 2, and 3 twice. Note that Module 3 is marked with a circle to highlight that it is the module analyzed in this work.

Visual and auditory stimuli were presented to the participants with precise timing control using OpenVIBE v1.3.0 software. The careful synchronization ensured accurate embedding of event markers within the EEG data stream, corresponding exactly to the occurrence of each stimulus. These markers were critical for aligning EEG signals precisely with cognitive events, enabling detailed and reliable ERP analyses.

In Module 1, the “Calm phase” (low workload), the participants maintained specified flight parameters without additional cognitive tasks, simulating minimal attentional demands.

In Module 2, the “Difficult phase” (moderate workload), the participants were required to respond to changing flight instructions issued at 30-s intervals, simulating a moderate cognitive load.

**Figure 4:**
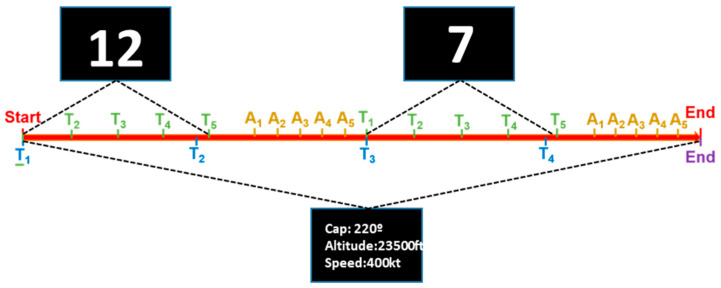
Module 3 timeline.

In Module 3, the “Arduous phase” (high workload), the participants simultaneously followed flight instructions at 45-s intervals and engaged in a Calculation and Memorization Task (CMT). During the CMT, numbers between 5 and 20 were presented at four-second intervals, each followed by an eight-second memorization period for cumulative arithmetic addition. After five numbers, the participants reported their computed sums in an answer phase prompted by auditory signals, with the responses compared against the correct answers displayed subsequently. Figure 4 shows the timeline for Module 3. The bottom of the line, from T1 to T4 (blue letters), shows the time at which the system changes the flight instruction. The top, from T1 to T5 (green letters), shows the time markers where the number appears on the screen and the pilot must perform the CMT task. Markers A1 to A5 (yellow letters) represent the time at which the system presents the CMT results, and the user checks whether they are correct or not.

This dual-task environment provided robust ERP signals indicative of high cognitive demands. Figure 2 shows a volunteer performing the CMT task inside the cockpit of the simulator in an experiment. The number to memorize is shown on the top left corner of the screen, and the flight instructions on the top right corner of the screen. The electrodes can be seen located on the head in the position listed in Section 2.2, and the reference for the EEG is also visible at the right lobe of the ear.

Module 3 was explicitly selected as the analytical focus of this study because it presents the highest cognitive demands within the experimental protocol. It is the only module that combines dynamic flight control instructions with a simultaneous arithmetic memorization task, both under strict temporal constraints. This dual-task configuration was specifically designed to replicate the types of multitasking and attentional challenges encountered in real-world aviation, such as managing instruments while processing mission-critical information. For this reason, Module 3 offers the most relevant environment for examining how event-related potentials, particularly the P300 component, reflect variations in mental workload.

As with all other modules in the protocol, Module 3 is subdivided into two consecutive segments, referred to as modules 3.1 and 3.2 (Figure 3). Each segment follows the same structure and task demands but occurs at a different point in time during the session. This internal organization allowed us to analyze how cognitive processing evolves over the course of sustained engagement. By comparing ERP responses between submodules 3.1 and 3.2, this study aimed to detect potential changes in P300 amplitude or latency that may reflect fatigue, adaptation, or shifts in cognitive strategy. The specific combination of high cognitive load and internal segmentation is what makes Module 3 uniquely suited for the in-depth ERP analysis presented in the following sections.

### 2.4. Dataset

This study compared the P300 elicited by two event types: impulse 0, marking the appearance of the number to be added, and impulse 255, indicating the correct answer. Thus, two distinct segmentation workflows were applied.

For impulse 0, all occurrences were retained for each participant and split into two files corresponding to modules 3.1 and 3.2, resulting in twenty-eight files. This allowed the direct assessment of training effects on the P300 at stimulus onset.

For impulse 255, each event was first labeled as correct or incorrect using a behavioral accuracy flag merged with the EEG event list. The data were then divided by accuracy and module, producing four subsets per participant (correct/incorrect × modules 3.1/3.2), yielding fifty-six files in total.

Unwanted events were removed in EEGLAB by selecting and deleting irrelevant impulses. In total, eighty-four condition-specific .set files were created (fourteen participants × six subsets), each aligned with the analytical comparisons defined earlier. These files served as input for the subsequent signal processing pipeline.

#### EEGLAB Processing

**Figure 5:**
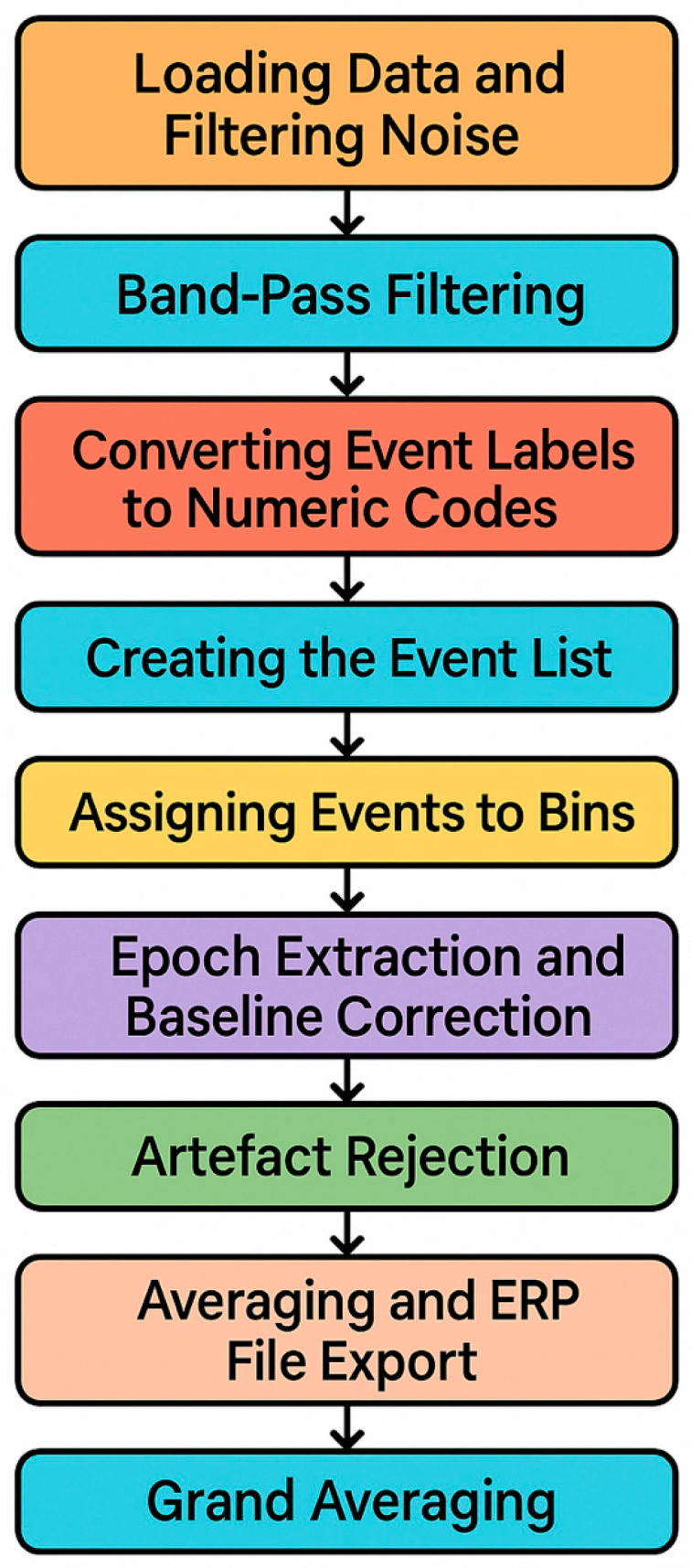
Data processing using EEGLAB.

EEG preprocessing was performed to convert 84 condition-specific raw datasets into high-quality ERP datasets suitable for statistical analysis. The preprocessing pipeline included line-noise removal, band-pass filtering, event marker recoding, epoch extraction, baseline correction, artefact rejection, averaging, and grand averaging (Figure 5).

Each dataset, corresponding to a single cognitive condition (anticipation or feedback), was processed individually. Line noise at 50 Hz was removed using a zero-phase notch filter, which preserved the temporal integrity of ERP waveforms. Continuous EEG was then band-pass filtered between 0.1 Hz and 15 Hz using a linear-phase FIR filter with a narrow transition band. This filter configuration attenuated slow drifts and high-frequency muscle artefacts while retaining the morphology of the P300 component, which was central to the analyses.

Event markers, initially encoded as strings, were converted into numeric codes and grouped into condition-specific categories (“bins”) corresponding to anticipation and feedback trials. This standardization ensured consistency across the participants and enabled condition-level averaging.

EEG epochs were extracted from −1000 ms to +2000 ms relative to stimulus onset. Baseline correction was applied using the −200 to 0 ms pre-stimulus interval to minimize slow drift effects. The data were re-referenced to the average of the mastoid electrodes, reducing lateral bias and improving comparability across the participants.

Artefact rejection combines automated detection and visual inspection. Epochs were excluded if the amplitude exceeded ±100 μV, if step-like changes surpassed 50 μV between consecutive samples, or if flat-line segments (<0.5 μV variance within 200 ms) indicated potential channel failure. A moving-window peak-to-peak procedure (200 ms window, 50 ms step) further identified transient artefacts. These thresholds were selected to maximize the retention of clean data while removing contaminated epochs.

Clean epochs were averaged within each bin to generate participant-level ERP waveforms. Finally, grand averages across the participants were computed for each condition, enhancing the signal-to-noise ratio and allowing robust analyses of P300 amplitude and latency across anticipation and feedback contexts.

### 2.5. Measurements and Metrics

To examine neural responses across the six experimental conditions, we computed grand average event-related potentials (ERPs) and extracted the P300 component, a well-established index of attention and cognitive processing [29]. Quantification of the P300 was based on two parameters: peak amplitude (in microvolts, μV) and peak latency (in milliseconds, ms), defined, respectively, as the maximum positive deflection and its corresponding time point within a predefined latency window.

Extraction was performed using the ERP Measurement Tool in ERPLAB version 10.4. Electrode sites Fz, Cz, and Pz were selected a priori based on their relevance to midline P300 distributions, with a particular emphasis on the centro-parietal maximum typically observed in oddball paradigms [30,31]. This electrode selection (Figure 1) enabled the analysis of both topographic variation and task-dependent modulations of the P300.

Each waveform was visually inspected in ERPLAB’s (v10.4) ERP Viewer to verify the presence and clarity of the P300 deflection within the expected latency range (typically 250–500 ms post-stimulus). In cases of ambiguous peak morphology, the measurement window was manually adjusted to ensure accurate peak detection. Amplitudes were calculated relative to a 200 ms pre-stimulus baseline. All extracted amplitude and latency values were exported in structured text format for statistical analysis in MATLAB R2023b. This systematic approach ensured temporal precision and reproducibility in the quantification of the P300 component, thereby supporting robust comparisons across experimental manipulations.

## 3. Results

This section presents the key results of the P300 changes elicited by two events, namely, anticipation of a new number (impulse 0) and feedback presentation (impulse 255), reflecting distinct cognitive processes. P300 amplitude and latency were analyzed across two task blocks (Modules 3.1 and 3.2) using data from electrodes Fz, Cz, and Pz. Statistical comparisons were performed using one-way ANOVA (*p* < 0.05), which was selected because of its suitability and widespread use in assessing mean differences across experimental conditions. Scalp topography maps were generated to illustrate spatial P300 distribution and changes in cortical activation related to stimulus type and repetition. The results are organized into three parts—two for each cognitive event and one for the scalp maps—each presenting measurements, statistical outcomes, and interpretations based on neurocognitive models.

### 3.1. P300 Answer to Impulse 0 (New Number Displayed)

**Figure 6:**
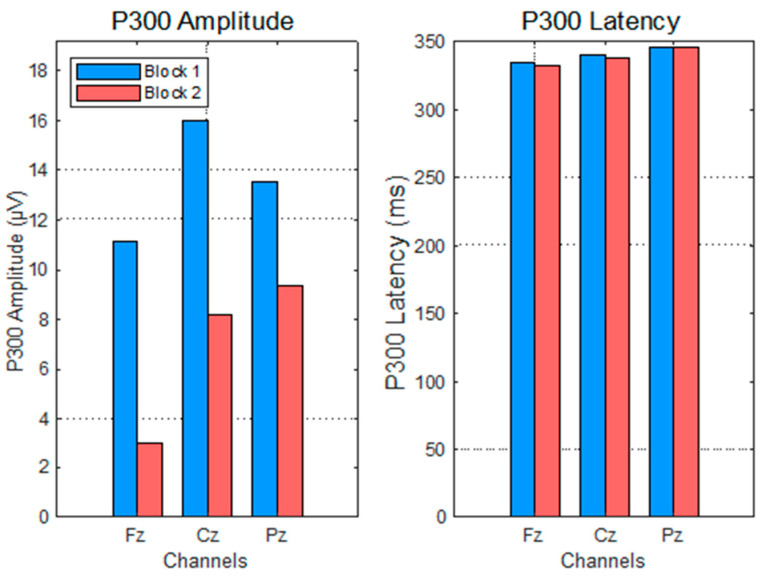
P300 evolution per electrode for impulse 0 (new number displayed).

P300 amplitude and latency were assessed at midline electrodes Fz, Cz, and Pz following the presentation of a new number (impulse 0), which marked the onset of a mental arithmetic task. Measurements were taken across two training blocks—Block 1 (submodule 3.1) and Block 2 (submodule 3.2)—to evaluate the effect of task repetition on electrophysiological responses. A consistent decrease in P300 amplitude was observed from Block 1 to Block 2 across all electrode sites. Specifically, the mean amplitude declined from 11.02 µV (SD = 4.33) to 2.85 µV (SD = 4.94) at Fz, from 15.91 µV (SD = 6.81) to 7.77 µV (SD = 5.88) at Cz, and from 13.21 µV (SD = 8.01) to 8.81 µV (SD = 6.53) at Pz (Figure 6).

**Figure 7:**
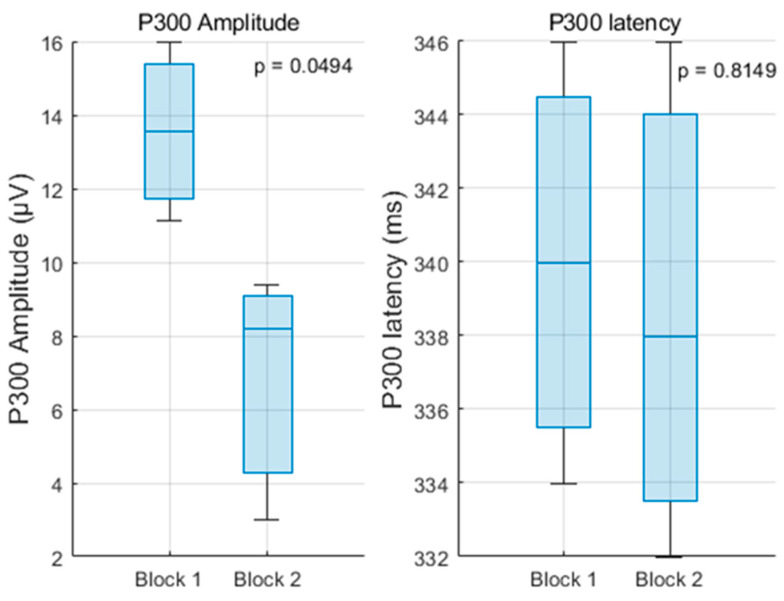
P300 evolution for impulse 0 (new number displayed)—ANOVA test.

A one-way repeated measures ANOVA confirmed that the reduction in P300 amplitude across the blocks was statistically significant (F(1,14) = 4.79, *p* = 0.0494, η^2^ = 0.25), indicating a medium effect size (Figure 7). In contrast, P300 peak latency did not exhibit significant variation across the two blocks. The mean latencies remained stable across electrodes, with values ranging from 332 to 345 ms in Block 1 and from 335 to 345 ms in Block 2.

The corresponding ANOVA yielded no statistically significant effect of block (F(1,14) = 0.06, *p* = 0.8149) (Figure 7). Together, these results suggest a decrease in neural resource allocation associated with repeated exposure to the task, as reflected by a reduced P300 amplitude. The unchanged latency, however, indicates that the temporal dynamics of stimulus evaluation remained constant across sessions, supporting the hypothesis that attentional engagement decreased with repetition, while processing speed was preserved.

### 3.2. P300 Response to Impulse 255 (Answer Display)

Impulse 255 corresponds to the presentation of the proposed result at the end of each trial and serves as external cognitive feedback. This section analyzes the P300 response elicited under correct and incorrect answer conditions, examining how feedback evaluation evolves with repeated exposure.

#### 3.2.1. Right Answers

In the case of correct answers, the displayed result confirms task success and acts as a reinforcement signal. The P300 elicited in this context reflects the evaluation of outcome relevance and can be modulated by factors such as task familiarity and confidence level.

**Figure 8:**
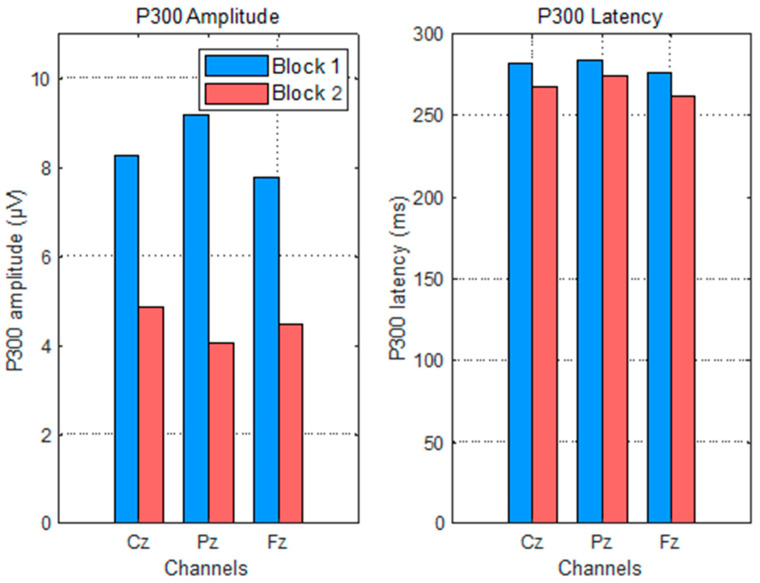
P300 evolution in amplitude and latency per electrodes Cz, PZ, and Cz, considering the right answers.

As shown in Figure 8, P300 amplitude decreased significantly from Block 1 to Block 2 across all midline electrodes (Cz: 8.3 → 4.9 µV; Pz: 9.3 → 4.1 µV; Fz: 7.8 → 4.6 µV). One-way ANOVA confirmed that this reduction was statistically significant (*p* = 0.0011; Figure 7). This amplitude decline suggests that the feedback became less cognitively salient with repetition, consistent with the context-updating theory [8], which posits that reduced novelty or motivational value leads to lower P300 responses. A possible contribution of cognitive fatigue due to sustained mental effort [32] cannot be excluded.

**Figure 9:**
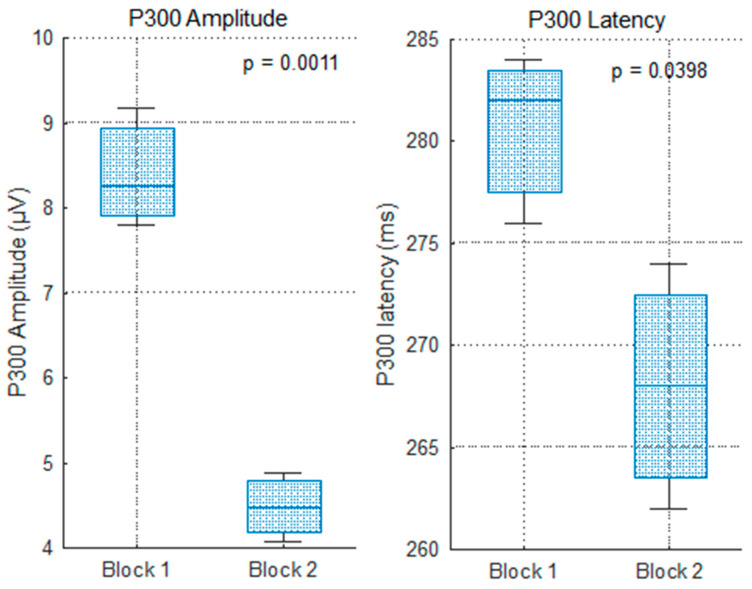
P300 evolution in amplitude and latency per electrodes Cz, PZ, and Cz, considering the right answers (Anovatest).

P300 latency also decreased slightly but significantly across the blocks (approx. 10–15 ms), as shown in Figure 8. This effect was confirmed by ANOVA (*p* = 0.0398; Figure 9), indicating a faster cognitive evaluation of correct feedback over time. Such reductions are commonly observed when task expectations stabilize and internal representations of outcomes become more efficient [33].

Together, the amplitude and latency results indicate both reduced attentional allocation and improved processing efficiency with task repetition. These effects are consistent with learning-related adaptations and support the sensitivity of the P300 to dynamic feedback evaluation processes [30].

#### 3.2.2. Wrong Answers

**Figure 10:**
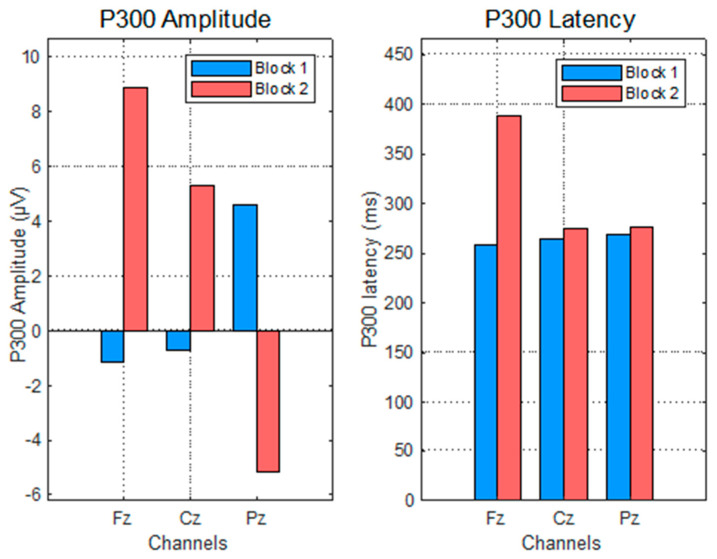
P300 evolution per electrode for impulse 255, considering the wrong answers (Answer Display).

The P300 response to wrong answers displayed high variability and lacked consistent topographic or temporal patterns. As shown in Figure 10, the amplitude values varied markedly between the blocks and electrodes, with negative peaks at Fz and Cz in Block 1 and large inconsistencies in Block 2.

**Figure 11:**
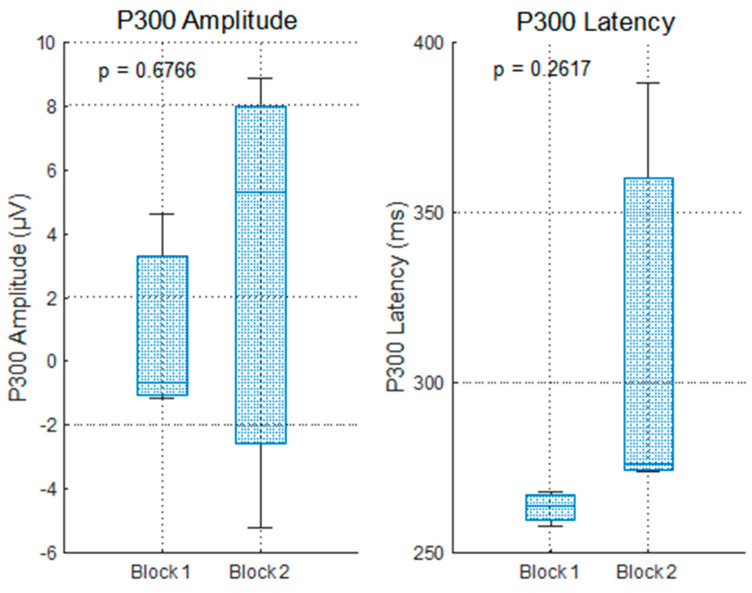
P300 evolution for impulse 255, considering wrong answers (Answer Display)—ANOVA test.

The ANOVA (Figure 11 revealed no significant effect of block on P300 amplitude (*p* = 0.6766), and the latency results were similarly nonsignificant (*p* = 0.2617). High inter-individual variability, particularly in Block 2, suggests insufficient statistical power due to the low number of error trials—a common limitation in ERP research when trial counts fall below reliability thresholds [34].

While the task design succeeded in eliciting correct responses, the scarcity of incorrect trials limited the robustness of the ERP averages, particularly for latency measures, which are highly sensitive to trial variability.

### 3.3. Scalp Topography of P300 Answers

To complement the statistical analysis of P300 amplitude and latency, scalp topography maps were generated using ERPLAB’s (v10.4) mapping tool. These maps visualize the spatial distribution of mean voltage across the scalp during the 250–400 ms window post-stimulus, a time range widely recognized as representative of the P300 component. This spatial perspective offers additional insight into neural activity patterns beyond individual electrode comparisons.

#### 3.3.1. Impulse 0—Anticipation Phase

**Figure 12:**
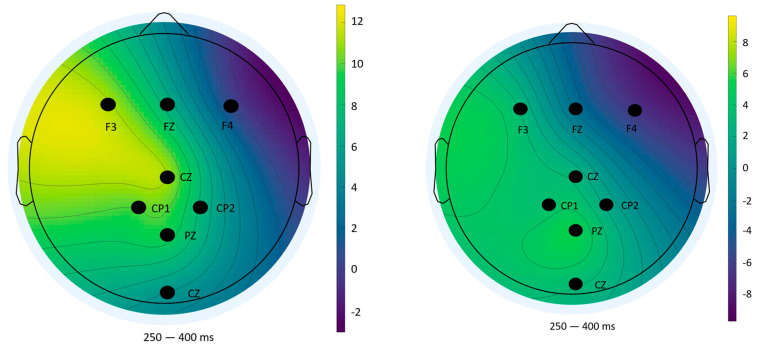
Topographical distribution of mean amplitude (250–400 ms) for impulse 0—Block 1 (**left**) and Block 2 (**right**).

Figure 12 depicts the topographic distribution of the mean P300 amplitude following the presentation of a new number (impulse 0) across Blocks 1 and 2. In Block 1, maximal activation was observed over central and slightly left-frontal regions, consistent with anticipatory processing demands. Although this deviates from the typical centro-parietal pattern reported in the literature [30,31], it remains compatible with a task-driven P300 distribution.

In Block 2, a marked reduction in amplitude was evident across the scalp, suggesting a decline in cognitive engagement, potentially reflecting habituation or accumulated cognitive fatigue [32].

#### 3.3.2. Impulse 255—Feedback Phase (Right Answers)

**Figure 13:**
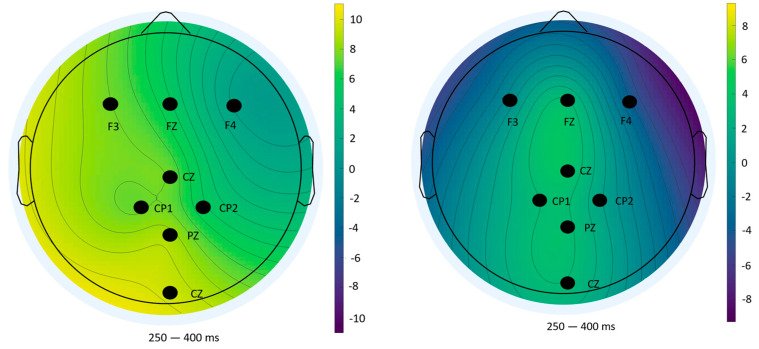
Topographical distribution of mean amplitude (250–400 ms) for impulse 255 (correct answers)—Block 1 (**left**) and Block 2 (**right**).

Considering the right answers to impulse 255, the topography in Block 1 (Figure 13 left) showed peak activation in left posterior parietal regions, a distribution consistent with feedback-evoked P300 responses [9].

In Block 2 (Figure 13 right), amplitude was globally reduced, reinforcing the interpretation of reduced stimulus salience and habituation effects [35,36].

#### 3.3.3. Impulse 255—Feedback Phase (Incorrect Responses)

**Figure 14:**
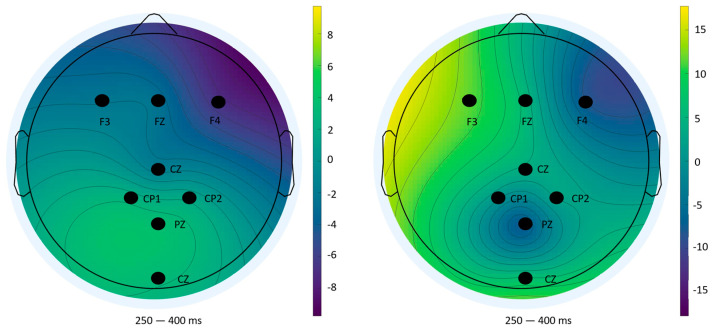
Topographical distribution of mean amplitude (250–400 ms) for impulse 255 (wrong answers)—Block 1 (**left**) and Block 2 (**right**).

In contrast, topographic maps for incorrect feedback (Figure 14) displayed high variability and lacked a consistent spatial pattern across blocks. This instability reflects the low number of error trials and aligns with the lack of significant statistical effects previously reported. Such variability reinforces the methodological limitations in ERP analysis under low trial count conditions [34].

Overall, the topographical analyses support the statistical findings and provide spatial confirmation of the cognitive effects observed—namely, reduced engagement and feedback salience across repeated exposure. These maps also highlight the differential neural dynamics associated with task anticipation and outcome evaluation.

## 4. Discussion

This study rigorously evaluated changes in P300 amplitude and latency across repeated blocks of a mental arithmetic task, differentiating between anticipation (impulse 0) and feedback processing (impulse 255) under correct and incorrect answer conditions.

The significant amplitude decreases in the P300 to impulse 0 after the first block support the notion of habituation and increased automaticity in attentional allocation to task onset stimuli. This decline aligns with cognitive theories positing that with practice, neural resource demands diminish as the task becomes more routine [9,37]. The stable latency further suggests that stimulus evaluation speed remains consistent despite reduced resource engagement.

For correct feedback, the simultaneous reduction in amplitude and latency implies more efficient neural processing of feedback information with learning. The amplitude decline likely reflects decreased novelty or surprise, while the latency shortening indicates accelerated stimulus categorization and evaluation processes [38,39]. This adaptive modulation aligns with prior findings in learning paradigms, where P300 dynamics mirror changes in expectancy and internal monitoring mechanisms [33].

The absence of clear P300 modulation for error feedback is predominantly attributed to the low frequency of incorrect trials, which compromises the ERP signal-to-noise ratio and impedes reliable component extraction. Future research should consider increasing task difficulty or trial numbers to ensure robust sampling of error events, enabling detailed examination of error-related negativity and feedback-related potentials [40,41].

Topographical analyses revealed spatial distributions consistent with established P300 generators: fronto-central areas during task anticipation and parietal sites during outcome evaluation [33,37]. The diminished amplitudes and altered distributions in Block 2 underscore habituation and resource reallocation, reinforcing the electrophysiological signatures of learning.

Statistical comparisons of P300 amplitude and latency across blocks and conditions were performed using one-way ANOVA (*p* < 0.05). ANOVA was chosen because our primary objective was to compare group means within a controlled experimental design, and it is a standard, widely accepted method in ERP research. Although no formal normality tests were conducted, the relatively balanced group sizes in our study, together with extensive empirical evidence from Monte Carlo simulations, support the robustness of the F-test to moderate violations of normality [42,43]. This provides confidence that the reported ANOVA results reliably reflect the underlying cognitive dynamics; however, we also acknowledge that future studies could complement this approach with non-parametric or Bayesian analyses to further validate the findings. Overall, the findings elucidate the nuanced dynamics of cognitive processing during repeated mental arithmetic tasks, highlighting the sensitivity of the P300 component to learning and adaptation processes. The differential patterns between anticipatory and evaluative stages underscore the multifaceted nature of attention and cognitive control reflected in ERP measures.

## 5. Conclusions

This study aimed to examine how the P300 component of event-related potentials (ERPs) reflects fluctuations in pilots’ cognitive states during complex flight scenarios characterized by elevated mental workload, induced through arithmetic memorization tasks. The analysis concentrated on two critical stimuli, namely, the onset of new numbers (impulse 0) and feedback presentation (impulse 255), with the objective of characterizing the temporal dynamics of attention allocation and stimulus evaluation under repeated cognitive demands.

The results unequivocally show that the P300 component is sensitive to changes in cognitive workload and the distribution of attentional resources. A consistent reduction in P300 amplitude was observed from the first to the second session across both stimulus types, indicating a progressive decline in attentional engagement, which likely reflects task habituation, increased familiarity, or the development of automaticity. Simultaneously, a significant decrease in P300 latency was found for correct feedback stimuli (impulse 255), suggesting enhanced efficiency in stimulus evaluation processes as exposure increased. In contrast, the latency for number onset stimuli (impulse 0) remained stable, implying that the fundamental speed of stimulus processing was maintained despite reduced attentional demands.

The responses to incorrect feedback stimuli were highly variable and did not exhibit statistically significant ERP effects, highlighting the need for sufficient trial numbers or greater task complexity to reliably capture neural correlates of infrequent and cognitively challenging events.

Collectively, these findings address the initial research question by demonstrating that P300 dynamics reliably index cognitive workload and shifts in attention during demanding flight-related tasks. The insights gained contribute to the human factors field by underscoring the potential of EEG-based cognitive monitoring to inform cockpit interface design and pilot training, thereby enhancing operational safety and efficiency in realistic aviation contexts.

Future investigations should consider augmenting this framework through the integration of continuous EEG measures, elevated task difficulty, or adaptive feedback mechanisms that respond in real-time to pilots’ cognitive states. In addition, complementary statistical approaches could be employed to further validate and refine the interpretation of EEG-derived metrics. For example, non-parametric or Bayesian analyses may provide more robust assessments in scenarios where assumptions underlying traditional ANOVA (normality, homogeneity of variance) are not fully met, and post hoc comparisons with appropriate multiple-comparison corrections could clarify specific sources of effects. Such advancements align with the overarching goal of embedding neuroergonomic biomarkers into next-generation cockpit systems and tailored training protocols, ultimately improving pilot performance and flight safety.

These findings further suggest that adaptive cockpit interfaces, which minimize extraneous cognitive load and emphasize critical information when attentional capacity is constrained, could yield substantial benefits. Moreover, EEG-guided training approaches may enable personalized adjustment of task demands to individual cognitive readiness, optimizing engagement and skill acquisition.

## Data Availability

The dataset, due to its sensitive nature, will only be provided upon a request that is considered reasonable by the authors.

## References

[1] Bruno M. (2021). Opération Poker.

[2] Endsley M.R. (1995). Toward a Theory of Situation Awareness in Dynamic Systems. Hum. Factors: J. Hum. Factors Ergon. Soc..

[3] Steven J.L. (2014). An Introduction to the Event-Related Potential Technique.

[4] Hillyard S.A., Anllo-Vento L. (1998). Event-related brain potentials in the study of visual selective attention. Proc. Natl. Acad. Sci. USA.

[5] Folstein J.R., Van Petten C. (2007). Influence of cognitive control and mismatch on the N2 component of the ERP: A review. Psychophysiology.

[6] Sutton S., Braren M., Zubin J., John E.R. (1965). Evoked-Potential Correlates of Stimulus Uncertainty. Science.

[7] Isreal J.B., Chesney G.L., Wickens C.D., Donchin E. (1980). P300 and Tracking Difficulty: Evidence For Multiple Resources in Dual-Task Performance. Psychophysiology.

[8] Donchin E., Coles M.G.H. (1988). Is the P300 component a manifestation of context updating?. Behav. Brain Sci..

[9] Polich J. (2007). Updating P300: An integrative theory of P3a and P3b. Clin. Neurophysiol..

[10] Sokhadze E.M., Casanova M.F., Casanova E.L., Lamina E., Kelly D.P., Khachidze I. (2017). Event-related Potentials (ERP) in Cognitive Neuroscience Research and Applications. NeuroRegulation.

[11] Ghani U., Signal N., Niazi I.K., Taylor D. (2020). ERP based measures of cognitive workload: A review. Neurosci. Biobehav. Rev..

[12] Wickens C., Kramer A., Vanasse L., Donchin E. (1983). Performance of Concurrent Tasks: A Psychophysiological Analysis of the Reciprocity of Information-Processing Resources. Science.

[13] Kramer A.F., Sirevaag E.J., Braune R. (1987). A Psychophysiological Assessment of Operator Workload During Simulated Flight Missions. Hum. Factors J. Hum. Factors Ergon. Soc..

[14] Sirevaag E.J., Kramer A.F., Reisweber C.D.W.M., Strayer D.L., Grenell J.F. (1993). Assessment of pilot performance and mental workload in rotary wing aircraft. Ergonomics.

[15] Fowler B. (1994). P300 as a Measure of Workload during a Simulated Aircraft Landing Task. Hum. Factors J. Hum. Factors Ergon. Soc..

[16] Natani K., Gomer F.E. Electrocortical Activity and Operator Workload: A Comparison of Changes in the Electroencephalogram and in Event-Related Potentials. https://apps.dtic.mil/sti/citations/ADA237188.

[17] Thiessen M.F., Lay J.E., Stern J.A. (1986). Neuropsychological Workload Test Battery Validation Study: Final Report on Air Force Contract.

[18] Wilson G.F., O’Donnell R.D. (1988). Measurement of Operator Workload with the Neuropsychological Workload Test Battery. Adv. Psychol..

[19] Wilson G.F., Eggemeier F.T. (2020). Psychophysiological Assessment of Workload in Multi-Task Environments. Multiple-Task Performance.

[20] Gratton G., Bosco C.M., Kramer A.F., Coles M.G., Wickens C.D., Donchin E. (1990). Event-related brain potentials as indices of information extraction and response priming. Electroencephalogr. Clin. Neurophysiol..

[21] Prinzel L.J., Pope A.T., Freeman F.G., Scerbo M.W., Mikulka P.J. (2007). Empirical Analysis of EEG and ERPs for Psychophysiological Adaptive Task Allocation.

[22] Aricò P., Borghini G., Di Flumeri G., Sciaraffa N., Babiloni F. (2018). Passive BCI beyond the lab: Current trends and future directions. Physiol. Meas..

[23] Hogervorst M.A., Brouwer A.-M., van Erp J.B.F. (2014). Combining and comparing EEG, peripheral physiology and eye-related measures for the assessment of mental workload. Front. Neurosci..

[24] Li H., Zhu P., Shao Q. (2024). Rapid Mental Workload Detection of Air Traffic Controllers with Three EEG Sensors. Sensors.

[25] Hankins T.C., Wilson G.F. (1998). A comparison of heart rate, eye activity, EEG and subjective measures of pilot mental workload during flight. Aviat. Space Environ. Med..

[26] Huizinga J.D., Chen J.-H. (2014). The myogenic and neurogenic components of the rhythmic segmentation motor patterns of the intestine. Front. Neurosci..

[27] Asensio-Cubero J., Gan J.Q., Palaniappan R. (2013). Multiresolution analysis over simple graphs for brain computer interfaces. J. Neural Eng..

[28] Parasuraman R., Sheridan T.B., Wickens C.D. (2008). Situation Awareness, Mental Workload, and Trust in Automation: Viable, Empirically Supported Cognitive Engineering Constructs. J. Cogn. Eng. Decis. Mak..

[29] Polich J., Criado J.R. (2006). Neuropsychology and neuropharmacology of P3a and P3b. Int. J. Psychophysiol..

[30] Picton T.W. (1992). The P300 Wave of the Human Event-Related Potential. J. Clin. Neurophysiol..

[31] Duncan C.C., Barry R.J., Connolly J.F., Fischer C., Michie P.T., Näätänen R., Polich J., Reinvang I., Van Petten C. (2009). Event-related potentials in clinical research: Guidelines for eliciting, recording, and quantifying mismatch negativity, P300, and N400. Clin. Neurophysiol..

[32] Ullsperger M., Freude G., Erdmann U. (2021). Auditory event-related potentials in severe burnout syndrome: Evidence for reduced cognitive brain resources. Int. J. Psychophysiol..

[33] Falkenstein M., Hoormann J., Hohnsbein J. (1999). ERP components in Go/Nogo tasks and their relation to inhibition. Acta Psychol..

[34] Luck S.J., Gaspelin N. (2016). How to get statistically significant effects in any ERP experiment (and why you shouldn’t). Psychophysiology.

[35] Kok A. (2001). On the utility of P3 amplitude as a measure of processing capacity. Psychophysiology.

[36] Umemoto A., Inzlicht M., Holroyd C.B. (2019). Electrophysiological indices of anterior cingulate cortex function reveal changing levels of cognitive effort and reward valuation that sustain task performance. Neuropsychologia.

[37] Donchin E. (1981). Surprise!? Surprise?. Psychophysiology.

[38] Verleger R. (1997). On the utility of P3 latency as an index of mental chronometry. Psychophysiology.

[39] Polich J., Luck S.J., Kappenman E.S. (2021). Neuropsychology of P300. The Oxford Handbook of Event-Related Potential Components.

[40] Miltner W.H.R., Braun C.H., Coles M.G.H. (1997). Event-Related Brain Potentials Following Incorrect Feedback in a Time-Estimation Task: Evidence for a “Generic” Neural System for Error Detection. J. Cogn. Neurosci..

[41] Holroyd C.B., Coles M.G.H. (2002). The neural basis of human error processing: Reinforcement learning, dopamine, and the error-related negativity. Psychol. Rev..

[42] Schmider E., Ziegler M., Danay E., Beyer L., Bühner M. (2010). Is it really robust? Reinvestigating the robustness of ANOVA against violations of the normal distribution assumption. Methodology.

[43] Blanca M.J., Alarcón R., Arnau J., Bono R., Bendayan R. (2017). Non-normal data: Is ANOVA still a valid option?. Psicothema.

